# Genetic alterations on chromosome 17p associated with response to radiotherapy in bulky cervical cancer

**DOI:** 10.1038/sj.bjc.6690658

**Published:** 1999-09

**Authors:** Y Harima, S Shirahama, K Harima, S Aoki, T Ohnishi, Y Tanaka

**Affiliations:** Department of Radiology, Kansai Medical University, Moriguchi, Osaka, 570-8506, Japan; Center for Molecular Biology and Cytogenetics, SRL, Inc., Hino, Tokyo, 191-0002, Japan; Department of Biology, Nara Medical University, Kashihara, Nara, 634, Japan

**Keywords:** loss of heterozygosity, microsatellite instability, radiotherapy, cervical carcinoma

## Abstract

Chromosome 17 alterations are found in more cancers than those of any other chromosome, and frequently involve the *p53* gene on 17p13. The aim of this study was to identify the correlations between the presence of loss of heterozygosity (LOH) and microsatellite instability (MI) on chromosome 17p13 in patients with cervical cancer and the patients’ response to radiotherapy. A total of 50 patients were treated with definitive radiotherapy. We performed biopsies and took specimens from the tumour and venous blood of all patients. Tumour and normal DNAs were analysed by polymerase chain reaction for genetic losses and instability at three polymorphic microsatellite loci mapped to 17p13. Nineteen of the 50 tumours (38%) displayed a genetic alteration (GA) on 17p13, 16 (32%) were found to have LOH, and three (6%) showed MI. The sizes of the tumours of the GA-positive patients were significantly greater than those of the GA-negative patients (*P* = 0.009). The mean tumour diameter of all patients was 6 ± 2.4 cm. We divided the patients into those with tumours smaller than 6 cm in diameter (*n* = 26) and those with tumours equal to or greater than 6 cm in diameter (*n* = 24). The former group survived significantly longer compared to the latter group (*P* = 0.0002). Among the patients with < 6 cm tumours, all six GA-positive patients are alive with no evidence of disease (NED), whereas of the 20 GA-negative patients, 18 have NED and two are alive with disease (AWD) or suffered cancer-caused death (CD). Thus, there was no correlation between GA and radiotherapy response in the tumours smaller than 6 cm. However, among the patients with ≥ 6 cm tumours, two of the GA-positive patients have NED and 11 are AWD/CD, whereas seven of the GA-negative patients have NED and four are AWD/CD. Among the patients with ≥ 6 cm tumours, the response to radiotherapy of the GA-positive patients were significantly poorer than those of the GA-negative patients (*P* = 0.02). In addition, the GA-negative patients survived significantly longer compared to the GA-positive patients (*P* = 0.026). The results of this study suggest that GA increases with tumour growth. Improved success in the management of bulky cervical cancer requires a better understanding of its biological behaviour. © 1999 Cancer Research Campaign


*British Journal of Cancer* (1999) **81**(1), 108–113
© 1999 Cancer Research Campaign
Article no. bjoc.1999.0658

### Genetic alterations on chromosome 17p associated with response to radiotherapy in bulky cervical cancer

**Y Harima[1], S Shirahama[2], K Harima[1], S Aoki[2], T Ohnishi[3] and Y Tanaka[1]**

[1]Department of Radiology, Kansai Medical University, 10-15 Fumizono-cho, Moriguchi, Osaka 570-8506, Japan; [2]Center for Molecular Biology and Cytogenetics, SRL, Inc., 5-6-50 Shinmachi, Hino, Tokyo 191-0002, Japan; [3]Department of Biology, Nara Medical University, 840 Shijo-cho, Kashihara, Nara 634, Japan

**Summary** Chromosome 17 alterations are found in more cancers than those of any other chromosome, and frequently involve the *p53* gene on 17p13. The aim of this study was to identify the correlations between the presence of loss of heterozygosity (LOH) and microsatellite instability (MI) on chromosome 17p13 in patients with cervical cancer and the patients' response to radiotherapy. A total of 50 patients were treated with definitive radiotherapy. We performed biopsies and took specimens from the tumour and venous blood of all patients. Tumour and normal DNAs were analysed by polymerase chain reaction for genetic losses and instability at three polymorphic microsatellite loci mapped to 17p13. Nineteen of the 50 tumours (38%) displayed a genetic alteration (GA) on 17p13, 16 (32%) were found to have LOH, and three (6%) showed MI. The sizes of the tumours of the GA-positive patients were significantly greater than those of the GA-negative patients ($P = 0.009$). The mean tumour diameter of all patients was 6 ± 2.4 cm. We divided the patients into those with tumours smaller than 6 cm in diameter ($n = 26$) and those with tumours equal to or greater than 6 cm in diameter ($n = 24$). The former group survived significantly longer compared to the latter group ($P = 0.0002$). Among the patients with < 6 cm tumours, all six GA-positive patients are alive with no evidence of disease (NED), whereas of the 20 GA-negative patients, 18 have NED and two are alive with disease (AWD) or suffered cancer-caused death (CD). Thus, there was no correlation between GA and radiotherapy response in the tumours smaller than 6 cm. However, among the patients with ≥ 6 cm tumours, two of the GA-positive patients have NED and 11 are AWD/CD, whereas seven of the GA-negative patients have NED and four are AWD/CD. Among the patients with ≥ 6 cm tumours, the response to radiotherapy of the GA-positive patients were significantly poorer than those of the GA-negative patients ($P = 0.02$). In addition, the GA-negative patients survived significantly longer compared to the GA-positive patients ($P = 0.026$). The results of this study suggest that GA increases with tumour growth. Improved success in the management of bulky cervical cancer requires a better understanding of its biological behaviour.

**Keywords:** loss of heterozygosity; microsatellite instability; radiotherapy; cervical carcinoma

Cervical cancer is the second leading cause of death from cancer in women worldwide (Pontén et al, 1995). Advances in surgical techniques, radiation therapy and the use of adjuvant chemotherapy have resulted in little improvement in the stage-by-stage cure rates of carcinoma of the cervix during the past 25 years (Kapp and Giaccia, 1996). Radiotherapy is the most important non-surgical treatment for cervical carcinoma. The tumour stage is still believed to be the most important determinant of prognosis in cervical carcinoma, and tumour size has also been used as a marker of response to radiotherapy and prognosis for many years (Virostek et al, 1996).

A better understanding of the molecular biology and radiobiology of cervical cancer will likely lead to the identification of new therapeutic interventions. Apoptosis plays an important role in the cytotoxic effects of radiation therapy (Harima et al, 1998) and chemotherapy (Lowe et al, 1994). The activation of the tumour suppressor gene (TSG) *p53* is associated with induction of apoptosis (Lowe et al, 1993). The biological behaviour of cervical cancer is determined by the nature of defects or mutations in key genes such as *p53*. However, in contrast to the situation in many other human tumours, *p53* mutations are only rarely detected in cervical cancers (Chang et al, 1995).

The inactivation or deletion of multiple TSGs, which otherwise regulate the normal cellular growth and suppression of abnormal cell proliferation, is recognized to be one of the major mechanisms of tumorigenesis. Mutations or deletions in a TSG are frequently accompanied by loss of the remaining allele, leading to homozygous inactivation of the gene (Marshall, 1991). Several studies have shown that loss of heterozygosity (LOH) at specific chromosomal sites is frequently associated with the development of various cancers, e.g. 3p14–p23 in small-cell lung carcinoma, 1p32–p36 in neuroblastoma, 13q14 in retinoblastoma, 5p21 in familiar adenomatous polyposis and 11p13–p15 in Wilms' tumour (Marshall, 1991). Although they have been relatively few, cytogenetic studies of cervical carcinoma have revealed frequent non-random chromosomal changes (Atkin et al, 1990). Studies of LOH in cervical carcinoma have also reported a high frequency of allelic deletions affecting 3p (Yokota et al, 1989; Jones et al, 1992; Kohno et al, 1993), 5p (Mitra et al, 1995), 6p (Mullokandov et al, 1996) and 11p (Srivatsan et al, 1991). LOH on 17p of cervical carcinoma has also been reported (Mullokandov et al, 1996; Jones et al, 1994; Mitra et al, 1994). Structural abnormalities of the short arm and loss of chromosome 17 have been reported to confer resistance to chemotherapy in patients with head and neck cancer (Li X

*Received 26 August 1998*
*Revised 8 February 1999*
*Accepted 16 February 1999*

*Correspondence to:* Y Harima et al, 1994), and in those with neuroblastoma (Caron et al, 1995). However, the importance of genetic alterations (GA) on chromosome 17p in the response of cervical cancer to radiotherapy remains unknown.

In this study, we evaluated the incidence of LOH and MI at 17p13 in the DNA of patients with cervical cancer, and assessed their impact on the outcome of radiotherapy.

### MATERIALS AND METHODS

### Patient characteristics

Between January 1995 and April 1998, 50 patients with histologically proven carcinoma of the uterine cervix (four stage Ib, five stage IIb, one stage IIIa, 30 stage IIIb, six stage IVa, four stage IVb) were treated with definitive radiotherapy at Kansai Medical University. The follow-up for the surviving patients ranged from 1 to 43 months, with a mean of 22.5 months. The mean patient age was 67.3 years (range 47–86 years). The primary tumours ranged in diameter from 1 to 11.9 cm (mean 6 cm), as measured by magnetic resonance imaging (MRI). Typical staging included a history, a physical examination, routine blood counts, a blood chemistry profile, a chest radiograph, an intravenous urogram, colonoscopy and computerized tomography (CT) and/or MRI. Patients were staged jointly by the radiation oncology and gynaecologic oncology staff according to the International Federation of Gynecology and Obstetrics (FIGO) classification (FIGO Annual Report, 1979), with modifications. The tumours consisted of 45 squamous cell carcinomas and five adenocarcinomas. No patient received chemotherapy prior to the radiotherapy. Each of the patients signed a consent form approved by the Kansai Medical University Review Board describing the experimental nature of the treatment.

### Irradiation techniques and doses

All patients entered in the protocol were treated with external pelvic radiation therapy using 6-MV high-energy linear accelerators. A total of 30.6 Gy was provided to the whole pelvis, plus an additional dose to the parametria with central shielding to complete 52.2 Gy, along with $^{192}$Ir high-dose-rate intracavitary brachytherapy. Radiation was delivered to the tumour in fractions of 1.8 Gy daily, 5 days per week. The dose of $^{192}$Ir brachytherapy, administered at a high dose rate, was 30 Gy to point A (point A being 2 cm lateral to the central canal of the uterus and 2 cm up from the mucous membrane of the lateral fornix, in the axis of the uterus) (ICRU Report 38, 1985), given as 7.5 Gy per session once per week.

### Detection and typing of HPV

Tumour samples were obtained from the 50 patients by punch biopsy prior to radiotherapy. Punch biopsies were taken from two to four different parts of each tumour and frozen immediately at –80°C. Genomic DNA was extracted from each tumour according to standard protocols (Bauer et al, 1992).

Tumour DNA was amplified by the polymerase chain reaction (PCR) with primers specific for human papillomavirus (HPV) 16, 18, 33 and 58 E6, as previously described (Yoshikawa et al, 1990). The PCR was carried out for 40 cycles at 95°C for 1.5 min, 48°C for 1.5 min and 70°C for 2 min, using a BioGene PHC-1 system (Techne, Cambridge, UK).

### Investigation of *p53* status

Mutations of the *p53* gene were identified by a single-strand conformation polymorphism (SSCP) analysis and DNA sequencing of tumour biopsies as previously described (Chen et al, 1993), using primers flanking the evolutionarily conserved regions of the gene from exon 5 to exon 8. Each exon was amplified separately using sense and antisense oligonucleotide primers flanking the exon as shown: exon 5, sense 5′-TTCCTCTTCCTGCAGTACTC-3′ and antisense 5′-GCCCCAGCTGCTCACCATCG-3′; exon 6, sense 5′-CACTGATTGCTCTTAGGTCTG-3′ and antisense 5′-AGTTGCAAACCAGACCTCAG-3′; exon 7, sense 5′-CCAAGGCGCACTGGCCTC-3′ and antisense 5′-GCGGCAAGCAGAGGCTGG-3′; exon 8, sense 5′-CCTATCCTGAGTAGTGGTAATC-3′, and antisense 5′-GTCCTGCTTGCTTACCTCGC-3′. All mutations were confirmed by sequencing or an SSCP analysis of a second independent PCR reaction. The products of this reaction were subcloned into TA vector (TA Cloning kit, Invitrogen). A mixture of at least 50 subclones was used as the template for DNA sequencing using a T7 sequencing kit (Boehringer Mannheim), visualized by exposure to Kodak XAR film with an intensifying screen at –80°C.

### Investigation of LOH and MI on 17p13.1

Regions of the genomic DNA extracted from the blood and tumour of each patient were amplified. PCR was utilized to detect the presence of LOH and MI on chromosome 17p with the three microsatellite markers D17S796, D17S1353 and D17S1881 (17p13.1). The PCR of each region was cycled 35 times at 94°C for 1 min, 55°C for 1 min, and 72°C for 1 min using each fluorescent set as previously described (Dib et al, 1996). The PCR products loaded on a 6% polyacrylamide gel were analysed by automatic sequencing (ALFred, Pharmacia; Uppsala, Sweden). Allelic losses were scored as decreases in intensity of one allele relative to the other as determined from a comparison of tumour and normal DNAs. MI was scored by comparing the electrophoretic pattern of the microsatellite markers amplified from the paired DNA specimen. The shift was indicated by either an addition or deletion of one or more repeat units resulting in the generation of novel microsatellite alleles. The analysis in the LOH- and MI-positive cases was repeated at least twice, and the results were highly reproducible.

### Statistical methods

Survival was measured as the time (days) from the date of the start of radiotherapy. The relationship between the presence/absence of genetic alterations on chromosome 17p13.1 and the radioresponse was analysed with Fisher's exact test. The tumour size data were analysed with a two-tailed Student's *t*-test. Actuarial survival was estimated by the Kaplan–Meier (1958) method, and differences in survival were analysed by the Breslow-Gehan-Wilcoxon test (Gehan, 1965). The statistical analyses were performed using Stata 4.0 Software (Stata Statistical Software: Release 4.0, Stata Corp., College Station, TX, USA). *P*-values below 0.05 were considered significant.

© 1999 Cancer Research Campaign

**Table 1** Clinicopathological characteristics of 50 patients with cervical carcinomas according to presence/absence of genetic alterations (GA) on chromosome 17p13.1

| | GA-positive tumours ($n$ = 19) | GA-negative tumours ($n$ = 31) | P |
|---|---|---|---|
| Age (years) | | | |
| Mean | 68.1 | 66.9 | NS[a] |
| SD | 10.6 | 8.5 | |
| Stage | | | |
| I | 0 | 4 | NS[b] |
| II | 1 | 4 | |
| III | 10 | 21 | |
| IV | 8 | 2 | |
| Histological type | | | |
| Squamous cell carcinoma | 17 | 28 | NS[b] |
| Adenocarcinoma | 2 | 3 | |
| Tumour size (cm) | | | |
| Mean | 7.1 | 5.3 | 0.009[a] |
| SD | 2.5 | 2.1 | |
| *p53* status | | | |
| Wild-type | 13 | 31 | 0.002[b] |
| Mutant-type | 6 | 0 | |
| HPV infection | | | |
| Positive | 6 | 13 | NS[b] |
| Negative | 13 | 18 | |

[a]Two-tailed Student's *t*-test; [b]Fisher's exact test.

**Table 2** Relationship between response to radiotherapy in all 50 patients: those with a tumour size smaller than 6 cm, greater than 6 cm and presence/absence of genetic alteration (GA) on chromosome 17p13

| | GA-positive tumours ($n$ = 19) | GA-negative tumours ($n$ = 31) | P |
|---|---|---|---|
| All cases ($n$ = 50) | | | |
| NED | 8 | 25 | 0.007 |
| AWD/CD | 11 | 6 | |
| < 6 cm ($n$ = 26) | | | |
| NED | 6 | 18 | NS |
| AWD/CD | 0 | 2 | |
| ≥ 6 cm ($n$ = 24) | | | |
| NED | 2 | 7 | 0.02 |
| AWD/CD | 11 | 4 | |

NED, no evidence of disease; AWD, alive with disease; CD, cancer death. *P*-value calculated by Fisher's exact test.

### RESULTS

Overall, 82% of the patients (41 of 50) had advanced disease. At present, 33 patients (66%) are alive and well (NED, no evidence of disease), five patients are alive with cancer disease (AWD) and 12 patients (24%) have died from recurrent disease (CD, cancer-caused death). In the patients with AWD and CD, eight patients have had a local failure, two have had distant metastases (lung in one patient, para-aortic lymph nodes in one), and seven have had both local failure and distant metastases (multiple metastases, six; lung, one).

### Relationships among stage, tumour size, response and patient outcome

There was a correlation between the stage of disease and the patients' response. All nine patients with stage I or II showed NED. In contrast, 17 of the 41 patients with advanced-stage cancer (i.e. stage III or IV) showed AWD or CD ($P$ = 0.02). The tumour sizes were 3.8 ± 2 cm in the stage I–II patients, 5.9 ± 1.9 cm in stage III, and 8.4 ± 2.2 cm in stage IV. The statistical analysis using the two-tailed Student's *t*-test revealed significant differences in tumour size between stage I–II and stage III ($P$ = 0.006), and stage I–II versus stage IV ($P$ = 0.0002). The tumour size increased with the stage of disease. At the time point at which the tumour size of the NED patients was 5 ± 2 cm, it had increased to 7.8 ± 2.7 cm in the AWD patients, and to 8 ± 1.5 cm in the CD patients. The difference in size was significant between the NED and AWD groups ($P$ = 0.008), and NED versus CD groups ($P$ = 0.00003, two-tailed Student's *t*-test). The mean tumour diameter of all patients was 6 ± 2.4 cm. We divided the patients into those with tumours smaller than 6 cm in diameter ($n$ = 26) and those with tumours equal to or greater than 6 cm in diameter ($n$ = 24). There was a significant difference in survival between these groups ($P$ = 0.0002, Breslow–Gehan–Wilcoxon test); the former group survived significantly longer than the latter group.

### The presence of HPV infection, stage, tumour size and outcome

HPV 16, 18, 33 or 58 genomes were detected by PCR in 19 patients (38%): in one stage I, four stage II, 11 stage III and three stage IV patients. The tumour size was 6.2 ± 2.5 cm in the HPV-positive tumours versus 5.9 ± 2.4 cm in the HPV-negative tumours. Of the HPV-positive patients, 13 have NED, four are AWD and two suffered CD. Of the HPV-negative patients, 20 have NED, one is AWD and ten suffered CD. Thus, there were no significant correlations between the tumour size (two-tailed Student's *t*-test), response (Fisher's exact test) or survival ($P$ = 0.12, Breslow–Gehan–Wilcoxon test) and HPV status.

### Relationships between p53 status and stage, tumour size and outcome

*p53* mutations were detected by SSCP analysis in six patients (12%): in five stage III patients and one stage IV patient. We observed *p53* mutations in codon 245, changing GGC to AGC, causing the substitution of serine for glysine; in codon 146, TGG to TAG, resulting in the substitution of a stop codon for tryptophane; in codon 189, GCC to GTC, causing the substitution of valine for alanine; and in codon 286, GAA to CAA, resulting in the substitution of glutamine for glutamic acid; and in codon 285, GAG to AAG, resulting in the substitution of lysine for glutamic acid; and in codon 213, CGA to TGA, resulting in the substitution of stop codon for arginine.

The tumour size was 8.2 ± 2.9 cm in the mutant *p53* tumours versus 5.7 ± 2.2 cm in the wild-type *p53* tumours; there was correlation between tumour size and *p53* status ($P$ = 0.02, two-tailed Student's *t*-test). Of the mutant *p53* patients, three have NED, one is AWD and two suffered CD. Of the wild-type *p53* patients, 30 have NED, four are AWD and ten suffered CD. There was no significant relationship between the NED group or AWD/CD group and *p53* status (Fisher's exact test). There was no significant

© 1999 Cancer Research Campaign

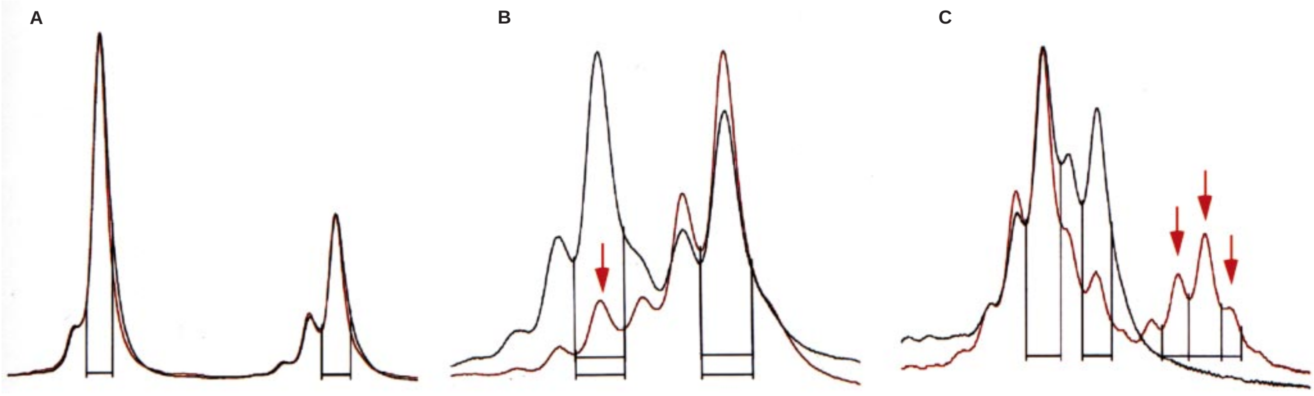

**Figure 1** Representative results of absence of genetic alteration (GA) (**A**), presence of loss of heterozygosity (LOH) (**B**), and presence of microsatellite instability (MI) (**C**) with the microsatellite marker D17S796 (17p13.1). The region was amplified of the genomic DNA extracted from blood (black line) and tumours (red line) of patients. The microsatellite alleles are represented by two peaks (heterozygotes). LOH (**B**) and MI (**C**) are indicated by red arrowheads

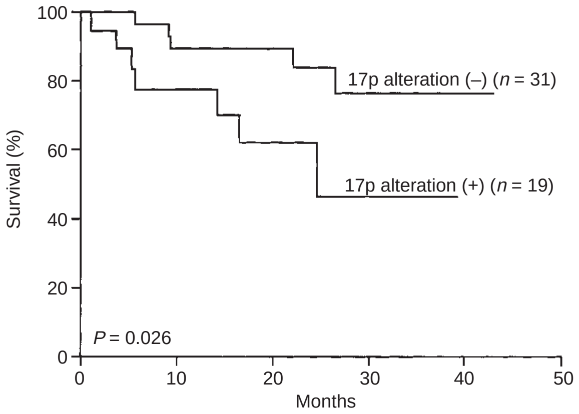

**Figure 2** Survival of patients with respect to presence/absence of genetic alteration (GA) on chromosome 17p13 using the Kaplan–Meier method and analysed by Breslow–Gehan–Wilcoxon test. The 31 GA-negative patients survived significantly longer compared to the 19 GA-positive patients ($P = 0.026$)

difference in survival between the wild-type *p53* patients and mutant *p53* patients ($P = 0.23$, Breslow–Gehan–Wilcoxon test).

### Relationships between GA on chromosome 17p13.1 and stage, tumour size and outcome

Nineteen of the 50 tumours displayed GA on 17p13.1 (38%), 16 (32%) showed LOH and three (6%) showed MI (Figure 1). The tumour size was 7.1 ± 2.5 cm in the GA-positive tumours, significantly larger than 5.3 ± 2.1 cm in the 31 GA-negative tumours ($P = 0.009$, two-tailed Student's *t*-test). LOH on 17p13.1 was seen in one stage II case, nine stage III, and six stage IV patients. MI on 17p13.1 was seen in one stage III patient and two stage IV patients. There was no correlation between the GA status and age, stage, histological type, or HPV infection. LOH was observed in all cases with mutant *p53*, but in none of the patients with MI. Thus, *p53* status was significantly related with the GA status ($P = 0.002$, Fisher's exact test, Table 1).

As shown in Table 2, of the 19 GA-positive patients, eight have NED (42.1%), four are AWD and seven showed CD. Of the 31 GA-negative patients, 25 have NED (80.6%), one is AWD and five showed CD. When we divided the patients into an NED group and an AWD/CD group, there was significant relationship between each of these groups and GA ($P = 0.007$, Fisher's exact test). We also evaluated the relationship between the two tumour-size groups and GA. In the group of patients with tumours smaller than 6 cm, all six of the GA-positive patients have NED, while 18 of the GA-negative patients have NED and two are AWD/CD. One of the patients with AWD had para-aortic lymph nodes metastases and another, with CD, had lung metastasis, although complete local control had been achieved in both patients. Thus, there was no correlation between GA and radiotherapy response in the tumours smaller than 6 cm. In the group of patients with tumours ≥ 6 cm, two of the 13 GA-positive patients have NED and 11 are AWD/CD. Of the GA-negative patients, seven have NED and four are AWD/CD. Thus, there was a significant relationship between GA and the radiotherapy response in the tumours 6 cm or greater ($P = 0.02$, two-tailed Student's *t*-test). In addition, among all patients, the GA-negative patients survived significantly longer compared to the GA-positive patients ($P = 0.026$, Breslow–Gehan–Wilcoxon test, Figure 2).

### DISCUSSION

The reported frequencies of LOH on 17p of cervical carcinoma vary considerably (between 15% and 35%) (Jones et al, 1994; Mitra et al, 1994; Mullokandov et al, 1996). In the present study, we investigated the correlation between response to radiotherapy and the incidence of LOH and MI at chromosome 17p in both tumour tissue specimens and blood of 50 patients with cervical carcinoma. In this study, LOH occurred in 32% of the tumours (16 of 50) and MI was revealed in 6% (three of 50). The patients with GA-positive tumours had significantly poorer responses ($P = 0.02$) and shorter survival ($P = 0.026$) compared to the GA-negative patients. These findings are consistent with results of studies of resistance to chemotherapy in patients with LOH on 17p in head and neck cancer (Li X et al, 1994) and in neuroblastoma (Caron et al, 1995).

Here, we report three patients with cervical cancer who showed MI in tumours on 17p13.1. Germline mutations in mismatch repair

© 1999 Cancer Research Campaign genes (*hMSH2*, *hMLH1*, *hPMS1* and *hPMS2*) (Fishel et al, 1993; Leach et al, 1993; Bronner et al, 1994; Nicolaides et al, 1994; Papadopoulos et al, 1994) cause a subset of familial colorectal cancers, termed hereditary non-polyposis colorectal cancer (HNPCC) (Lynch et al, 1993). As a consequence of such mutations, errors arising during DNA replication are less efficiently corrected. However, the relationship between MI and cervical carcinomas remains unclear. With the exception of one report (Mitra et al, 1995), there is no evidence of MI in cervical carcinomas. In the report by Mitra et al (1995), LOH and MI were studied only in the 5p14-ter region, and replication error-type alterations were found in six (13%) of 46 invasive carcinomas, two (40%) of five cases of carcinoma in situ (CIS) and three (21%) of 14 precancerous lesions. In our study, we found MI in the 17p13 region in three cervical cancer patients, one at stage III, two at stage IVb. None of these three tumours were infected HPV or harboured mutant-*p53*. We suspect that cervical tumours with genetic instability might have other alterations related to mismatch repair system genes, such as *hMLH1* and *hMSH2*.

The importance of the *p53* gene in clinical oncology has been reviewed (Chang et al, 1995). A poor response of human malignancies to different therapies is often associated with mutations of the *p53* gene, such as in breast cancer (Aas et al, 1996) and colon cancer (Ahnen et al, 1998). In contrast to the situation in many other human tumours, *p53* mutations are only rarely detected in cervical cancers (Chang et al, 1995). In the present study also, only six of the 50 patients (12%) were found to have mutations in the *p53* gene, as evaluated by an SSCP analysis of genomic DNA. The six tumours with mutant *p53* had G:C to A:T mutations. These data are in agreement with a report on the high frequency of G:C to A:T mutations in cervical carcinoma (Park et al, 1994).

Most cervical carcinomas have been shown to contain HPV DNA sequences, including the high risk-HPV 16 and HPV 18 types (zur Hausen and de Villier, 1994). The binding of high risk-HPV types E6 viral protein to p53 protein has been shown to result in a rapid ubiquitin-dependent proteolytic degradation of p53 (Scheffner et al, 1990). Therefore, the presence of high-risk forms of HPV is believed to result in a loss of the *p53*-mediated control of cell growth (Butz et al, 1995). In general, the presence of high-risk HPV in tumour cells is thought to be associated with a poor prognosis in cervical cancer treated with radical hysterectomy (Burger, 1996). It has been shown, however, that HPV-positive cancer cells may preserve the p53 protein in its functional form (Butz et al, 1995). This observation may partially explain discrepancies in the results of studies examining the correlation between the presence of various types of HPV DNA in tumours and treatment outcome in cervical cancer (King et al, 1989; Higgins et al, 1991). In the case of cervical carcinoma, there is an obvious need to study the effects of HPV infection on intrinsic tumour cell radiosensitivity (Kapp and Giaccia, 1996). However, the literature on differences in prognosis between HPV-positive and HPV-negative tumours is incomplete (Kristensen et al, 1996; Burger et al, 1996). No correlations were found between HPV infection and treatment outcome in our study.

E6 type-specific PCR was more sensitive than L1 consensus PCR (Iwasaka et al, 1996), therefore we evaluated HPV status using type-specific E6 primers. In this study, 38% of the patients (19 of 50) were HPV-positive. There are three explanations for this low rate of HPV-positive tumours. At first, Matsumoto et al (1997) reported that HPV-positive tumours were detected in 49% of cervical cancers obtained from Japanese women. In the second, Kawana (1995) reported that HPV-DNA was detected more frequently in the cases of cervical dysplasia than among those of cervical cancer in Japan. Due to the high rate (82%) of advanced-stage patients in this study, the low rate of HPV-positive tumours might have occurred. In the third, the mean age of patients was 67.3 years (range 47–86 years) in our study. At the same time, the prevalence of HPV in western Europe and USA is generally less than 10% at 40 years of age or older (Schiffman et al, 1992).

In this study, tumour size was found to be the most important determinant of response to radiotherapy and prognosis for cervical carcinoma, which is in agreement with previous reports (Virostek et al, 1996). There was no correlation between the response and GA in the tumours smaller than 6 cm. In contrast, among the patients with tumours ≥ 6 cm, the response of the patients with GA-positive tumours was significantly poorer than that of the GA-negative patients. The results of this study suggest that GAs on 17p are important predictors of outcome following radiation treatment in patients with bulky cervical carcinomas. The higher incidence of GA in tumours or greater than 6 cm may explain, at least in part, the poor prognosis of this category of patients.

The analysis of genetic instability in patients with advanced cervical carcinomas might help to clarify further the mechanisms of the rapid progression and poor treatment response in some individuals. In turn, such analysis will contribute essential knowledge for the development of new strategies for cancer gene therapy.

### ACKNOWLEDGEMENTS

This study was supported by a Grant-in-Aid for Scientific Research from the Ministry of Education, Science, Sports and Culture, Japan.

### REFERENCES

Aas T, Børresen AL, Geisler S, Smith-Sørensen B, Johnsen H, Varhaug JE, Akslen LA and Lønning PE (1996) Specific p53 mutations are associated with de novo resistance to doxorubicin in breast cancer patients. *Nat Med* **2**: 811–814

Ahnen DJ, Feigl P, Quan G, Fenoglio Preiser C, Lovato LC, Bunn PA Jr, Stemmerman G, Wells JD, Macdonald JS and Meyskens FL Jr (1998) Ki-ras mutation and p53 overexpression predict the clinical behaviour of colorectal cancer: a Southwest Oncology Group study. *Cancer Res* **58**: 1149–1158

Atkin NB, Baker MC and Fox MF (1990) Chromosome changes in 43 carcinomas of the cervix uteri. *Cancer Genet Cytogenet* **44**: 229–241

Bronner CE, Baker SM, Morrison PT, Warren G, Smith LG, Lescoe MK, Kane M, Earabino C, Lipford J, Lindblom A, Tannergård P, Bollag RJ, Godwin AR, Ward DC, Nordenskjøld M, Fishel R, Kolodner R and Liskay RM (1994) Mutation in the DNA mismatch repair gene homologue hMLH1 is associated with hereditary non-polyposis colon cancer. *Nature* **368**: 258–261

Burger RA, Monk BJ, Kurosaki T, Anton Culver H, Vasilev SA, Berman ML and Wilczynski SP (1996) Human papillomavirus type 18: association with poor prognosis in early stage cervical cancer. *J Natl Cancer Inst* **88**: 1361–1368

Butz K, Shahabeddin L, Geisen C, Spitkovsky D, Ullmann A and Hoppe-Seyler F (1995) Functional p53 protein in human papillomavirus-positive cancer cells. *Oncogene* **10**: 927–936

Caron H (1995) Allelic loss of chromosome 1 and additional chromosome 17 material are both unfavourable prognostic markers in neuroblastoma. *Med-Pediatr-Oncol* **24**: 215–221

Chang F, Syrjänen S and Syrjänen K (1995) Implications of the p53 tumor-suppressor gene in clinical oncology. *J Clin Oncol* **13**: 1009–1022

Chen TM, Chen CA, Hsieh CY, Chang DY, Chen YU and Defendi V (1993) The state of p53 in primary human cervical carcinomas and its effects in human papillomavirus-immortalized human cervical cells. *Oncogene* **8**: 1511–1518

Dib C, Faure S, Fizames C, Samson D, Drouot N, Vignal A, Millasseau P, Marc S, Hazan J, Seboun E, Lathrop M, Gyapay G, Morissette J and Weissenbach J (1996) A comprehensive genetic map of the human genome based on 5, 264 microsatellite. *Nature* **380**: 152–154

© 1999 Cancer Research Campaign

FIGO (1979) Annual Report on the Results of Treatment in Carcinoma of the Uterus, Vagina, and Ovary, vol. 16, FIGO: Radiumhemmet, Sweden
Fishel R, Lescoe MK, Rao MR, Copeland NG, Jenkins NA, Garber J, Kane M and Kolodner R (1993) The human mutator gene homolog MSH2 and its association with hereditary nonpolyposis colon cancer. *Cell* **75**: 1027–1038
Gehan EA (1965) A generalized Wilcoxon test for comparing arbitrarily singly-censored samples. *Biometrika* **52**: 203–223
Harima Y, Harima K, Shikata N, Oka A, Ohnishi T and Tanaka Y (1998) Bax and Bcl-2 expressions predict response to radiotherapy in human cervical cancer. *J Cancer Res Clin Oncol* in press
Higgins GD, Davy M, Roder D, Uzelin DM, Philips GE and Burrell CJ (1991) Increased age and mortality associated with cervical carcinomas negative for human papillomavirus RNA. *Lancet* **338**: 910–913
ICRU Report No. 38 (1985) Dose and Volume Specification for Reporting Intracavitary Therapy in Gynecology. International Commission of Radiation Units and Measurements: Bethesda, MD.
Iwasaka A, Nieman P, Lehtinen M and Paavonen J (1996) Human papillomavirus DNA in uterine cervix squamous cell carcinoma and adenocarcinoma detected by polymerase chain reaction. *Cancer* **77**: 2275–2279
Jones MH and Nakamura Y (1992) Deletion mapping of chromosome 3p in female genital tract malignancies using microsatellite polymorphisms. *Oncogene* **7**: 1631–1634
Jones MH, Koi S, Fujimoto I, Hasumi K, Kato K and Nakamura Y (1994) Allelotype of uterine cancer by analysis of RFLP and microsatellite polymorphisms: frequent loss of heterozygosity on chromosome arms 3p, 9q, 10q, and 17p. *Genes Chromosomes Cancer* **9**: 119–123
Kaplan EL and Meier P (1958) Non-parametric estimation from incomplete observations. *J Am Stat Assoc* **53**: 457–481
Kapp DS and Giaccia A (1996) New direction for radiation biology research in cancer of the uterine cervix. *J Natl Cancer Inst* **21**: 131–138
Kawana T (1995) Human papilloma virus and cervical cancer. *Jpn J Cancer Chemother* **22**: 711–717
King LA, Tase T, Twiggs LB, Okagaki T, Savage JE, Adcock LL, Prem KA and Carson LF (1989) Prognostic significance of the presence of human papillomavirus DNA in patients with invasive carcinoma of the cervix. *Cancer* **63**: 897–900
Kohno T, Takayama H, Hamaguchi M, Takano H, Yamaguchi N, Tsuda H, Hirohashi S, Vissing H, Shimizu M, Oshimura M and Yokota J (1993) Deletion mapping of chromosome 3p in human uterine cervical cancer. *Oncogene* **8**: 1825–1832
Kristensen GB, Karlsen F, Jenkins A, Kaern J, Abeler VM and Trope CG (1996) Human papilloma virus has no prognostic significance in cervical carcinoma. *Eur J Cancer* **32**: 1349–1353
Leach FS, Nicolaides NC, Papadopoulos N, Liu B, Jen J, Parsons R, Peltomaki P, Sistonen P, Aaltonen LA, Nyström-Lahti M, Guan XY, Zhang J, Meltzer PS, Yu JW, Kao FT, Chen DJ, Cerosaletti KM, Fournier REK, Todd S, Lewis T, Leach RJ, Naylor SL, Weissenbach J, Mecklin JP, Järvinen H, Petersen GM, Hamilton SR, Green L, Jass J, Watson P, Lynch HT, Trent JM, Chapelle A, Kinzler KW and Vogelstein B (1993) Mutations of a mutS homolog in hereditary nonpolyposis colorectal cancer. *Cell* **75**: 1215–1225
Li X, Lee NK, Ye YW, Waber PG, Schweitzer C, Cheng QC and Nisen PD (1994) Allelic loss at chromosomes 3p, 8p, 13q, and 17p associated with poor prognosis in head and neck cancer. *J Natl Cancer Inst* **86**: 1524–1529
Lowe SW, Schmitt EM, Smith SW and Osborne BA (1993) p53 is required for radiation–induced apoptosis in mouse thymocytes. *Nature* **362**: 847–849
Lowe SW, Bodis S, McClatchey A, Remington L, Ruley HE, Fisher DE, Housman DE and Jacks T (1994) P53 status and the efficacy of cancer therapy in vivo. *Science* **266**: 807–810
Lynch HT, Smyrk TC, Watson P, Lanspa SJ, Lynch JF, Lynch PM, Cavalieri RJ and Boland CR (1993) Genetics, natural history, tumor spectrum, and pathology of hereditary nonpolyposis colorectal cancer: an updated review. *Gastroenterology* **104**: 1535–1549
Marshall CJ (1991) Tumor suppressor genes. *Cell* **64**: 313–326
Matsumoto K, Yoshikawa H, Taketani Y, Yoshiike K and Kanda T (1997) Antibodies to human papillomavirus 16, 18, 58, and 6b major capsid proteins among Japanese females. *Jpn J Cancer Res* **88**: 369–375
Mitra AB, Murty VV, Li RG, Pratap M, Luthra UK and Chaganti RS (1994) Allelotype analysis of cervical carcinoma. *Cancer Res* **54**: 4481–4487
Mitra AB, Murty VV, Singh V, Li RG, Pratap M, Sodhani P, Luthra UK and Chaganti RS (1995) Genetic alterations at 5p15: a potential marker for progression of precancerous lesions of the uterine cervix. *J Natl Cancer I* **87**: 742–745
Mullokandov MR, Kholodilov NG, Atkin NB, Burk RD, Johnson AB and Klinger HP (1996) Genomic alterations in cervical carcinoma: losses of chromosome heterozygosity and human papilloma virus tumor status. *Cancer Res* **56**: 197–205
Nicolaides NC, Papadopoulos N, Liu B, Wei YF, Carter KC, Ruben SM, Rosen CA, Haseltine WA, Fleischmann RD, Fraser CM, Adams MD, Venter JC, Dunlop MG, Hamilton SR, Petersen GM, Chapelle A, Vogelstein B and Kinzler KW (1994) Mutations of two PMS homologues in hereditary nonpolyposis colon cancer. *Nature* **371**: 75–80
Papadopoulos N, Nicolaides NC, Wei YF, Ruben SM, Carter KC, Rosen CA, Haseltine WA, Fleischmann RD, Fraser CM, Adams MD, Venter JC, Hamilton SR, Petersen GM, Watson P, Lynch HT, Peltomäki P, Mecklin JP, Chapelle A, Kinzler KW and Vogelstein B (1994) Mutation of a mutL homolog in hereditary colon cancer. *Science* **263**: 1625–1629
Park DJ, Wilczynski SP, Paquette RL, Miller CW and Koeffler HP (1994) p53 mutations in HPV-negative cervical carcinoma. *Oncogene* **9**: 205–210
Srivatsan ES, Misra BC, Venugopalan M and Wilczynski SP (1991) Loss of heterozygosity for alleles on chromosome 11 in cervical carcinoma. *Am J Hum Genet* **49**: 868–877
Scheffner M, Werness BA, Huibregtse JM, Levine AJ and Howley PM (1990) The E6 oncoprotein encoded by human papillomavirus types 16 and 18 promotes the degradation of p53. *Cell* **63**: 1129–1136
Schiffman MH (1992) Recent progress in defining the epidemiology of human papillomavirus infection and cervical neoplasia. *J Natl Cancer Inst* **84**: 394–398
Pontén J, Adami HO, Bergström R, Dillner J, Friberg LG, Gustafsson L, Miller AB, Parkin DM, Sparén P and Trichopoulos D (1995) Strategies for global control of cervical cancer. *Int J Cancer* **60**: 1–26
Virostek LJ, Kim RY, Spencer SA, Meredith RF, Jennelle RL, Soong SJ and Salter MM (1996) Postsurgical recurrent carcinoma of the cervix: reassessment and results of radiation therapy options. *Radiology* **201**: 559–563
Yokota J, Mori N, Akiyama T, Shimosato Y, Sugimura T and Terada M (1989) Multiple genetic alterations in small-cell lung carcinoma. *Princess Takamatsu Symp* **20**: 43–48
Yoshikawa H, Kawana T, Kitagawa K, Mizuno M, Yoshikura H and Iwamoto A (1990) Amplification and typing of multiple cervical cancer-associated human papillomavirus DNAs using a single pair of primers. *Int J Cancer* **45**: 990–992
zur Hausen H and E de Villier (1994) Human papillomavirus. *Annu Rev Microbiol* **48**: 427–447

© 1999 Cancer Research Campaign 
